# Regulation of Tight Junctions in Upper Airway Epithelium

**DOI:** 10.1155/2013/947072

**Published:** 2012-12-29

**Authors:** Takashi Kojima, Mitsuru Go, Ken-ichi Takano, Makoto Kurose, Tsuyoshi Ohkuni, Jun-ichi Koizumi, Ryuta Kamekura, Noriko Ogasawara, Tomoyuki Masaki, Jun Fuchimoto, Kazufumi Obata, Satoshi Hirakawa, Kazuaki Nomura, Takashi Keira, Ryou Miyata, Nobuhiro Fujii, Hiroyuki Tsutsumi, Tetsuo Himi, Norimasa Sawada

**Affiliations:** ^1^Department of Pathology, Sapporo Medical University School of Medicine, Sapporo 060-8556, Japan; ^2^Department of Otolaryngology, Sapporo Medical University School of Medicine, Sapporo 060-8556, Japan; ^3^Department of Pediatrics, Sapporo Medical University School of Medicine, Sapporo 060-8556, Japan; ^4^Department of Microbiology, Sapporo Medical University School of Medicine, Sapporo 060-8556, Japan

## Abstract

The mucosal barrier of the upper respiratory tract including the nasal cavity, which is the first site of exposure to inhaled antigens, plays an important role in host defense in terms of innate immunity and is regulated in large part by tight junctions of epithelial cells. Tight junction molecules are expressed in both M cells and dendritic cells as well as epithelial cells of upper airway. Various antigens are sampled, transported, and released to lymphocytes through the cells in nasal mucosa while they maintain the integrity of the barrier. Expression of tight junction molecules and the barrier function in normal human nasal epithelial cells (HNECs) are affected by various stimuli including growth factor, TLR ligand, and cytokine. In addition, epithelial-derived thymic stromal lymphopoietin (TSLP), which is a master switch for allergic inflammatory diseases including allergic rhinitis, enhances the barrier function together with an increase of tight junction molecules in HNECs. Furthermore, respiratory syncytial virus infection in HNECs *in vitro* induces expression of tight junction molecules and the barrier function together with proinflammatory cytokine release. This paper summarizes the recent progress in our understanding of the regulation of tight junctions in the upper airway epithelium under normal, allergic, and RSV-infected conditions.

## 1. Introduction

**Figure 1:**
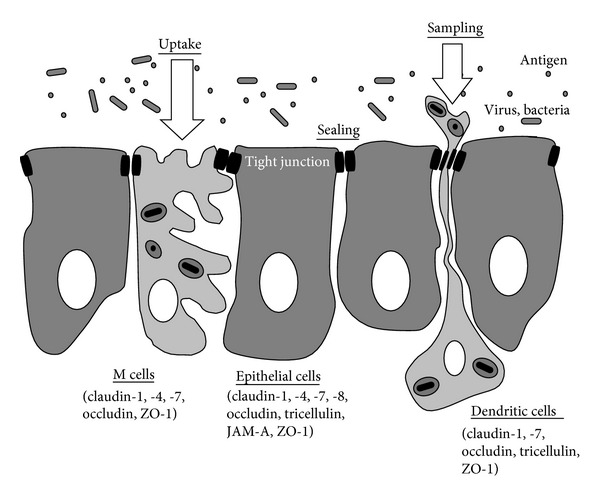
Schema of putative sealing intercellular spaces by tight junction molecules in the upper airway epithelium including epithelial cells, M cells, and dendritic cells.

The epithelium in the upper respiratory consists of pseudostratified ciliated columnar epithelial cells, including M cells (membranous or microfold cells), which are specialized for antigen uptake and form a continuous barrier against a wide variety of exogenous antigens [1–5], and dendritic cells (DCs), which take up transported antigens via M cells and present antigens for CD4^+^ T cells, while they maintain the integrity of the airway epithelial barrier [6–8]. The epithelium plays a crucial role as an interface of adaptive responses and innate responses via tight junctions to prevent invasion of inhaled environmental agents such as allergens and pathogens (Figure 1). In addition, in the human nasal mucosa of allergic rhinitis or virus infection, dynamic changes of tight junctions have been known.

## 2. Tight Junctions in Epithelium

The airway epithelium of the human upper respiratory mucosa acts as the first physical barrier that protects against inhaled substances and pathogens [9, 10]. The epithelium is a highly regulated and impermeable barrier exclusively formed by tight junctions [9, 10].

Tight junctions, the most apically located of the intercellular junctional complexes, inhibit solute and water flow through the paracellular space (termed the “barrier” function) [11, 12]. They also separate the apical from the basolateral cell surface domains to establish cell polarity (termed the “fence” function) [13, 14]. Recent evidence suggests that tight junctions also participate in signal transduction mechanisms that regulate epithelial cell proliferation, gene expression, differentiation, and morphogenesis [15]. 

Tight junctions are formed by not only the integral membrane proteins claudins, occludin, and JAMs, but also many peripheral membrane proteins, including the scaffold PDZ expression proteins zonula occludens (ZO)-1, ZO-2, ZO-3, multi-PDZ domain protein-1 (MUPP1), and membrane-associated guanylate kinase with inverted orientation-1 (MAGI)-1, MAGI-2, MAGI-3, and cell polarity molecules ASIP/PAR-3, PAR-6, PALS-1, and PALS-1 associated tight junction (PATJ), and the non-PDZ-expressing proteins, cingulin, symplekin, ZONAB, GEF-H1, aPKC, PP2A, Rab3b, Rab13, PTEN, and 7 H6 [16–18]. Zonula occludens-1 (ZO-1), ZO-2, and ZO-3 are members of the membrane-associated guanylate kinase (MAGUK) family of proteins displaying a characteristic multidomain structure comprised of SH3, guanylate kinase-like (GUK), and multiple PDZ (PSD95-Dlg-ZO1) domains [19]. ZO-1 and ZO-2 are also closely associated with polymerization of claudins [20]. Tricellulin is first identified at tricellular contacts where there are three epithelial cells and is shown to have a barrier function [21]. More recently, lipolysis-stimulated lipoprotein receptor (LSR) is found as a tricellular tight junction-associated membrane protein and recruits tricellulin to tricellular tight junctions [22].

The claudin family, which consists of at least 27 members, is solely responsible for forming tight junction strands and has four transmembrane domains and two extracellular loops [17, 23]. The first extracellular loop is the coreceptor of hepatitis C virus [24] and influences the paracellular charge selectivity [25], and the second extracellular loop is the receptor of *Clostridium perfringens* enterotoxin (CPE) [26]. In addition, as claudin-4 is also a high-affinity receptor of CPE [27], full-length CPE with a direct cytotoxic effect and the C-terminal receptor binding domain of CPE (C-CPE) without a cytotoxic effect are employed for selective treatment and drug delivery against claudin-4 expressing cells [28, 29]. 

Occludin, the first discovered integral membrane protein of tight junctions, is most ubiquitously expressed at the apicalmost basolateral membranes and is the most reliable immunohistochemical marker for tight junctions [17, 30]. Overexpression of occludin increases the barrier function, indicated as an increase in transepithelial electric resistance (TER) increase in mammalian epithelial cells [31, 32]. However, TJ strands can be formed without occludin in some cell types, including occludin-deficient embryonic stem cells [33, 34]. Moreover, an occludin-deficient mouse model does not display a perturbation of epithelial barrier function, although a complex pathophysiological phenotype is observed with growth retardation, chronic inflammation and hyperplasia of the gastric epithelium, calcification in the brain, testicular atrophy, loss of cytoplasmic granules in striated duct cells of the salivary gland, and thinning of the compact bone [35].

JAMs (JAM-A, -B, -C, -4) are immunoglobulin superfamily proteins expressed at cell junctions in epithelial and endothelial cells as well as on the surfaces of leukocytes, platelets and erythrocytes [36]. They are important for a variety of cellular processes, including tight junction assembly, leukocyte transmigration, platelet activation, angiogenesis, and adenovirus binding. Current evidence indicates that JAM-A dimerization is necessary for functional regulation of barrier [37].

## 3. Expression and Localization of Tight Junction Molecules and Its Function in the Epithelium of the Upper Respiratory *In Vivo* and *In Vitro*

**Figure 2:**
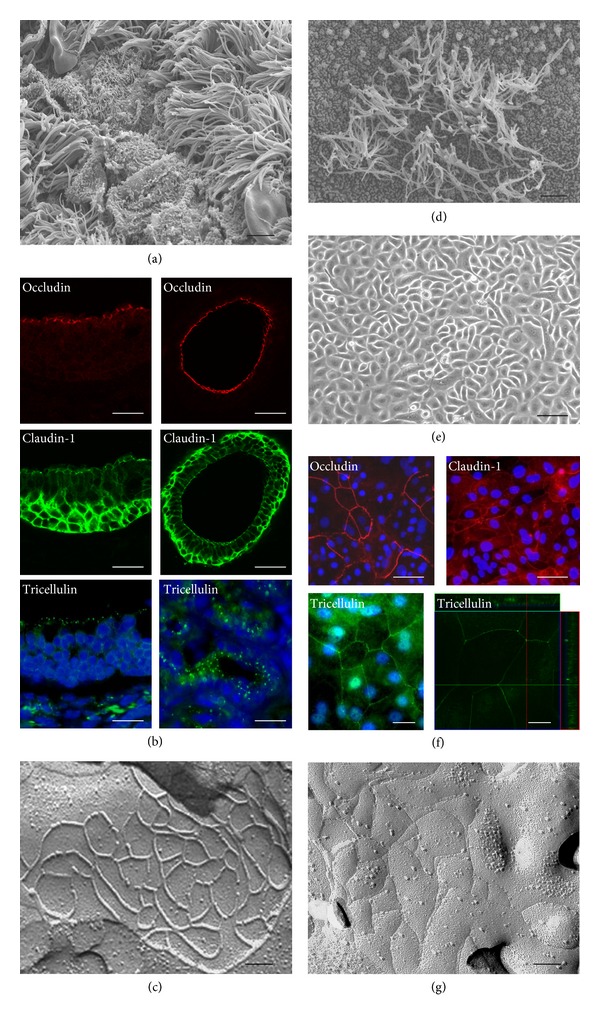
SEM image (a), immunostaining (b) for occludin, claudin-1 and tricellulin, and freeze-fracture image (c) in human nasal mucosa *in vivo*. Scanning electron microscopy (SEM) image (d), phase contrast (e), immunostaining (f) for occludin, claudin-1 and tricellulin, and freeze-fracture image (g) in human nasal epithelial cells *in vitro* (hTERT-HNECs). Scale bars: (a) and (d) = 800 nm, (b) and (f) = 10 *μ*m, (c) and (g) = 200 nm, and (e) = 20 *μ*m.

**Figure 3:**
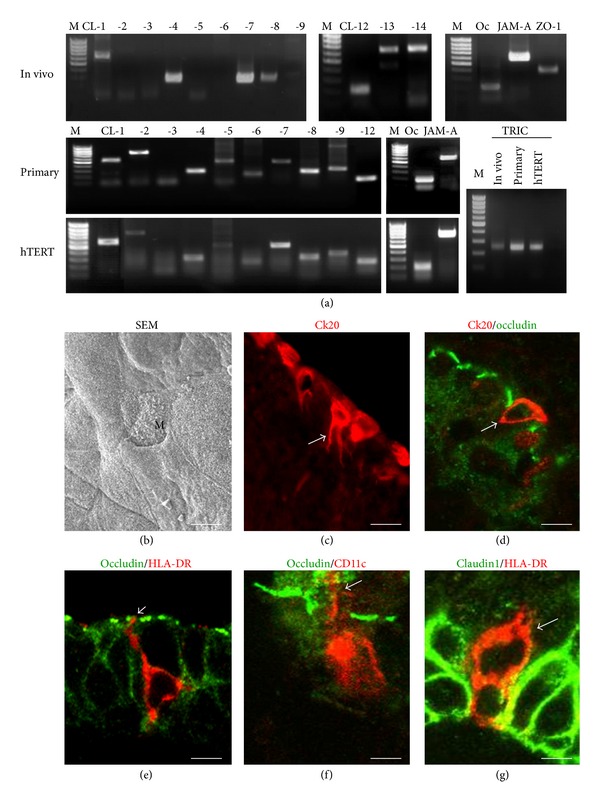
RT-PCR (a) for mRNAs of tight junction molecules in human nasal mucosa *in vivo* and two types of human nasal epithelial cells *in vitro* (primary and hTERT-HNECs). M, 100-bp ladder DNA marker; Oc, occludin; CL, claudin. SEM image (b) and immunostaining for Ck20 (c), Ck20, and occludin (d) in human adenoidal epithelium *in vivo*. Immunostaining for occludin and HLA-DR (e), occludin and CD11c (f), and claudin-1 and HLA-DR (g) in human nasal epithelium *in vivo*. M: M-like cell. Scale bars: (a) = 20 *μ*m, (b) = 5 *μ*m, (c) and (d) = 20 *μ*m, and (e)–(g) = 10 *μ*m.

We investigated the expression and localization of tight junction molecules and its function in the epithelium of the upper respiratory *in vivo* and *in vitro* (Figures 2 and 3) [38–44]. 

In human nasal mucosa *in vivo* in which was observed many cilia on the surface, mRNAs of occludin, JAM-A, ZO-1, ZO-2, claudin-1, -4, -7, -8, -12, -13, -14, and tricellulin are detected [38, 41]. Occludin, JAM-A, and ZO-1 were found in the uppermost layer. Claudin-1 was observed in the uppermost and basal layers in the epithelium. Claudin-4 and -7 were observed throughout the epithelium. In freeze-fracture replicas, continuous lines of tight junction strands formed well-developed networks on the subapical membranes. 

It is known that the proliferation and storage of epithelial cells in primary cultures are very limited. We introduced the catalytic component of telomerase, the hTERT gene, into primary cultured human nasal epithelial cells [39]. The ectopic expression of hTERT in the epithelial cells resulted in greater growth potential and a longer lifespan of the cells. The cells had a small cobblestone appearance in phase-contrast images. The cilia-like structures, a differentiation marker of nasal epithelial cells, were observed on the surface of hTERT-transfected cells (Figure 1). The properties of the passaged hTERT-transfected cells were similar to those of the cells in primary cultures [39].

We investigated tight junctions in hTERT-HNECs compared to primary cultured cells [39, 40]. In both hTERT-HNECs and primary cultured cells, mRNAs of claudin-1, -2, -4, -5, -6, -7, -8, -9, and -12, occludin, and JAM-A were detected. Claudin-1, -4, occludin, and JAM-A were observed at cell borders cells. The continuous lines of tight junction strands formed well-developed networks on the subapical membranes. In HNECs *in vitro* using primary cultures and our established culture systems, tight junction molecules and the barrier function are upregulated by various stimuli (Table 1). The hTERT-transfected human nasal epithelial cells (hTERT-HNECs) can be used as an indispensable and stable model for studying regulation of tight junctions in human nasal epithelium.

## 4. Tight Junctions of M Cells in the Epithelium of the Upper Respiratory

M cells (membranous or microfold cells) are known as specialized epithelial cells of the follicle-associated epithelium (FAE), and the role seems to be the rapid uptake of particular antigens and microorganisms to the immune cells of the lymphoid follicle to induce an effective immune response [45]. The structure and functional peculiarities of M cells are observed in different species and at different sites of the lymphoid tissue along the digestive and respiratory tracts [46]. The respiratory M cells act as a nasopharynx-associated lymphoid tissue in the upper respiratory tract [3]. Furthermore, claudin-1, -3, and ZO-1 are detected in M cells of mouse intestinal follicle-associated epithelium (FAE) [47].

The adenoidal epithelium, including M cells, which are specialized for antigen uptake, forms a continuous barrier against a wide variety of exogenous antigens [48]. Several markers such as lectin histochemistry and immunoreactivity to vimentin, cytokeratins, and annexin-V have been proposed to identify M cells in the mouse, rat, hamster, rabbit, and pig [45, 46, 49–51]. In the nasopharyngeal tonsil of the horse, lectin GS-1 B4 (Griffonia simplicifolia 1 isolectin B4) has been used as a marker for M cells [52]. It was reported that cytokeratin20 (Ck20) could serve as an M cell marker for rabbit palatine tonsils [53]. In humans, clusterin is expressed in M cells and follicular dendritic cells at inductive sites of human mucosa-associated lymphoid tissue [54]. Class II beta-tubulin is a specific histochemical marker for human tonsillar M cells and follicular dendritic cells [55]. However, universal markers for human M cells have not yet been established. 

We identified M-like cells using an anti-Ck20 antibody in human adenoidal tissues *in vivo* and *in vitro* and investigated the expression of tight junctions (Figure 3) [5]. In human adenoidal epithelium *in vivo*, some M-like cells, characterized by irregular microvilli, were observed on the surface. Some Ck20-positive cells were randomly observed in the epithelium and appeared as pocket-like structures. In both Ck20-positive and -negative cells of the adenoidal epithelium *in vivo*, occludin, ZO-1, claudin-1, and -7 were observed. In the primary cultures, Ck20-positive cells took up fluorescent microparticles *in vitro*. In both Ck20-positive and -negative cells of primary cultures *in vitro*, occludin, ZO-1, claudin-1, and -7 were clearly observed at cell borders. These findings indicated that M cells in the adenoid could take up antigens while maintaining a continuous barrier.

## 5. Tight Junctions of Dendritic Cells in the Epithelium of the Upper Respiratory 

This function requires DCs, professional antigen-presenting cells that act as peripheral sentinels specializing in the uptake, processing, and presentation of antigenic material.

Rescigno et al. discovered a new mechanism for pathogen uptake in the mucosa by which DCs open the tight junctions between epithelial cells and send dendrites outside the epithelium to directly sample the pathogen. DCs express tight junction proteins such as occludin, claudin-1, and ZO-1 to preserve the integrity of the epithelial barrier [56]. 

The epithelial DC population expresses high levels of the Langerhans cell (LC) marker langerin and the tight junction proteins claudin-1, -7, and ZO-2 [57]. Claudin-1 is detected in murine CD207^+^ LCs residing in the epidermis but not in other skin DCs [58]. In human THP-1 monocytes, mRNAs of occludin, tricellulin, JAM-A, ZO-1, ZO-2, and claudin -4, -7, -8, and -9 can be detected. In mature DCs that have dendrites elongated by treatment with IL-4, GM-CSF, TNF-*α*, and ionomycin, mRNA and protein of JAM-A are significantly increased compared to monocytes [59]. We previously reported that in mouse XS52 DCs, claudin-1, -3, -4, -6, -7, -8, and occludin are detected and claudin-7 is induced via an NF-*κ*B pathway by thymic stromal lymphopoietin (TSLP) and ligands of Toll-like receptor 2 (TLR2), TLR4, or TLR7/8 [60].

In the human nasal mucosa of allergic rhinitis, HLA-DR-, and CD11c-positive DCs express tight junction protein claudin-1 and penetrate beyond the apicalmost tight junction protein occludin to minimize the increase in permeability of the epithelial barrier (Figure 3) [38, 61].

## 6. TLR3 Ligand Reduced Tight Junctions in the Epithelium of the Upper Respiratory

TLRs are a component of the innate immune system [62, 63]. They enable the host to recognize a large number of pathogen-associated molecular patterns such as those of bacterial lipopolysaccharides (LPS), viral RNA, CpG-containing DNA, and flagellin, among others [64]. TLRs are also expressed in the epithelium of the upper respiratory and may play a vital role in the immunological outcomes in these tissues, which produce proinflammatory cytokines and chemokines upon ligation [65]. 

In human nasal epithelium *in vivo* and *in vitro*, mRNAs for all 10 known human TLRs are detected *in vivo* and *vitro* [66]. TLR3 recognizes viral double-stranded (dsRNA) and its synthetic analogue polyinosinic-polycytidylic acid (poly(I:C)) and stimulates innate immune responses [67]. In primary cultures of human adenoid epithelial cells that expressed mRNAs of TLR1, 2, 3, 4, 6, 7, and 10, stimulation by the TLR3 ligand poly(I:C), induced production of not only TNF*α* and IL-8 but also reduced JAM-A expression. The changes were regulated via distinct signaling transduction pathways [44]. 

The control of TLR3-mediated signaling pathways in human nasal epithelium may be important not only in infection by viral dsRNA but also in autoimmune diseases caused by endogenous dsRNA released from necrotic cells.

## 7. TSLP Induced Tight Junctions in the Epithelium of the Upper Respiratory

The epithelial-derived factor TSLP is an IL-7-like cytokine that potently induces deregulation of Th2 responses, a hallmark feature of allergic inflammatory diseases such as asthma, atopic dermatitis, and allergic rhinitis [42, 68–70]. TSLP-stimulated CD11c^+^ DCs induce naïve CD4^+^ T cells to differentiate into Th2 cells that produce IL-4, IL-5, IL-13, and TNF-*α* [69]. We found high expression of TSLP in epithelium from patients with allergic rhinitis with recruitment and infiltration of CD11c^+^ DCs [42]. *In vitro*, TSLP was significantly produced in HNECs by treatment with a TLR2 ligand Pam_3_Cys-Ser-(Lys)_4_ and a mixture of IL-1*β* and TNF-*α*. Treatment with TSLP rapidly enhanced the barrier function of cultured HNECs together with an increase of tight junction proteins claudin-1, -4, -7, and occludin. The nasal epithelial-derived TSLP not only activates DCs but also preserves the epithelial barrier via upregulation of tight junction proteins to regulate antigen sensitization during the early stage of allergic rhinitis. 

## 8. The Effects of Lymphocytes on Tight Junctional Barrier of the Upper Respiratory

 Chronic rhinosinusitis (CRS) is characterized by mucosal inflammation involving both the nasal cavity and paranasal sinuses [71]. The patients with chronic rhinosinusitis and nasal polyps have a Th2-predominant type of inflammation [72]. The leaky epithelium is present *in vivo* and *in vitro* in patients with downregulation of claudin-4 and occludin mRNA in the biopsy specimens [73]. Furthermore, the barrier function in human primary sinonasal epithelial cells is decreased by the Th1 cytokine IFN-*γ* and Th2 cytokine IL-4, whereas Th17 cytokine has no effect [73].

 On the other hand, B lymphocytes which are responsible for the production of IgE play a crucial role in allergic and inflammatory of upper and lower airways [74]. Nasal polyps have increased numbers of activated eosinophils, mast cells, and IgE [75]. Nasal polyp epithelium from human tissue specimens has reduced claudin-1 along the basal aspect of the mucosal layer, whereas occludin is reduced in the apical and basal epithelial zones [76]. However, the closed relationship between B cells and tight junctional barrier remains still unknown.

## 9. RSV Induced Tight Junctions in the Epithelium of the Upper Respiratory

The airway epithelium, which has a well-developed barrier regulated by tight junctions, is the first line of defense during respiratory virus infection. Moreover, it is also known that tight junctions include targets or receptors of viruses such as claudin-1 and occludin as coreceptors of HCV, JAM as a reovirus receptor, and CAR as a coxsackie and adenovirus receptor [77]. In human nasal epithelial cells, rhinovirus infection decreases expression of tight junction molecules ZO-1, occludin, and claudin-1 and reduced barrier function using primary cultures [78].

**Figure 4:**
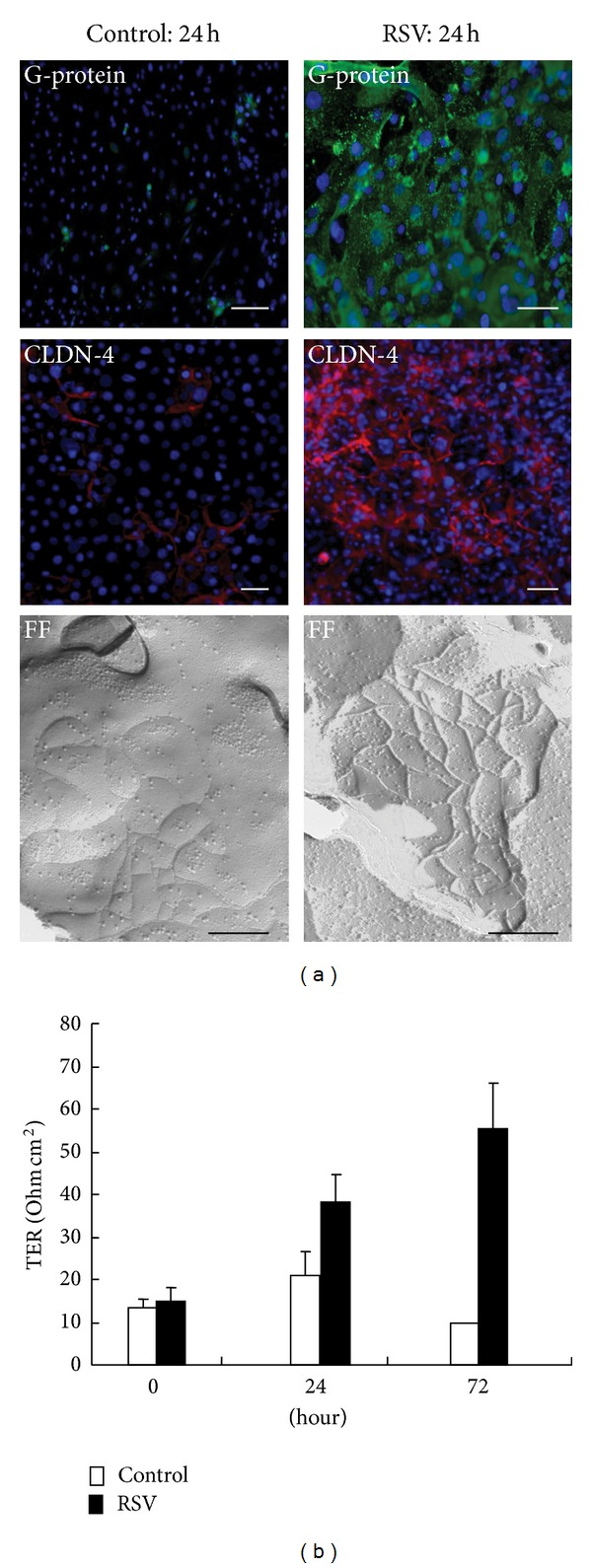
(a) Phase contrast, immunostaining for RSV/G-protein and claudin-4, and freeze-fracture image in human nasal epithelial cells *in vitro* (hTERT-HNECs) at 24 h after infection with RSV. Scale bars: white bars = 20 *μ*m, black bars = 100 nm. (b) Transepithelial electrical resistance (TER) values in hTERT-HNECs at 24 h after infection with RSV. *N* = 3, *P < 0.01 versus control.

On the other hand, respiratory syncytial virus (RSV) is the major cause of bronchitis, asthma, and severe lower respiratory tract disease in infants and young children [79]. To investigate the detailed mechanisms of replication and budding of RSV in human nasal epithelial cells (HNECs) and the epithelial cell responses including tight junctions, we established an RSV-infected model using hTERT-transfected HNECs [80]. When the HNECs were infected with RSV at MOI 1, the expression of RSV/G-proteins was detected in most cells by immunocytochemistry using a specific antibody (Figure 4). We found that the expression, structure and barrier function of tight junction molecules claudin-4 and occludin were markedly induced together with production of proinflammatory cytokines IL-8 and TNF*α* in HNECs after RSV infection, and the induction of tight junction molecules possibly contributed to budding of RSV (Figure 4) [80]. However, the knockdown of claudin-4 and occludin by siRNAs did not affect replication of RSV. The replication and budding of RSV and the epithelial cell responses in HNECs were regulated via a PKC*δ*/HIF-1*α*/NF-*κ*B pathway [80]. The control of this pathway in HNECs may be useful not only for prevention of replication and budding of RSV but also in therapy for RSV-induced respiratory pathogenesis.

## 10. Effects of C-CPE via Claudins in the Epithelium of the Upper Respiratory

COOH-terminal half fragment of CPE (C-CPE) is a nontoxic molecule that disrupts tight junction barrier function and enhances cellular absorption [81]. More recently, by screening claudin-binders from a C-CPE mutant-displaying library by using claudin-displaying budded baculovirus, a C-CPE mutant called m19, which bound to claudin-1, -2, -4, and -5, is made [82]. In our recent study, when HNECs were treated with wild type and m19 of C-CPE, the barrier function was markedly decreased at the nontoxic concentration of C-CPE and recovered without C-CPE (personal data). 

## 11. Conclusions

The epithelial tight junction barrier of the upper airway epithelium, including M cells and DCs, is stably maintained via the regulation of tight junction molecules expressed in the epithelial cells, M cells, and DCs. Various antigens are sampled, transported, and released to lymphocytes through the cells while maintaining the integrity of the barrier (Figure 1). 

Claudin-4 is known as a rodent M cell-specific gene and the high transcytotic ability of M cells is an attractive target for mucosally delivered vaccines and therapeutics [83, 84]. Furthermore, C-CPE which is a nontoxic small molecule that disrupts tight junction barrier function can be used as a carrier for other substances to specific claudin-positive cells [81]. These may promote development of a novel strategy for drug delivery system via targeted claudins in upper airway epithelium.

Tricellular tight junctions form at the convergence of bicellular tight junctions where three epithelial cells meet in polarized epithelia, and tricellulin was the first marker of the tricellular tight junction identified in epithelial cells [21]. In various DCs, including human THP-1 cells, mouse XS52 cells, and epidermal LCs, tricellulin is also detected [59, 60]. The tricellular tight junction may be a good penetration point for DCs into the epithelium [85]. More recently, it is reported that *Shigella* targets tricellular junctions including tricellulin to spread between cells via noncanonical clathrin-dependent endocytic pathway [86]. Thus, further study of tricellulin in epithelial cells and DCs of upper airway epithelium may be important to prevent invasion of inhaled environmental agents such as allergens and pathogens.

Taken together, these studies of tight junctions in upper airway epithelium should provide new insights not only into pathological conditions but also in the context of new vaccines and therapeutics against infectious and inflammatory mucosal diseases in upper airway.

## Figures and Tables

**Table 1:** Changes of tight junction proteins and barrier function in HNECs *in vitro*.

Treatments		Tight junction proteins	Barrier function
FBS		CLDN-1↑; CLDN-4↑	Upregulation
Growth factor	TGF-*β*	CLDN-4↑	No change
PKC activator	TPA	CLDN-1↑; OCDN↑; ZO-1↑; ZO-2↑	Upregulation
PPAR*γ* ligands	Rosiglitazone Troglitazone	CLDN-1↑; CLDN-4↑; OCDN↑; TRIC↑ CLDN-1↑; CLDN-4↑; OCDN↑	Upregulation
TLR3 ligand	Poly I:C	JAM-A↓	No change
Cytokine	TSLP	CLDN-1↑; CLDN-4↑; CLDN-7↑; OCDN↑	Upregulation
Virus	RSV	CLDN-4↑; OCDN↑	Upregulation
